# Serum BPIFB4 levels classify health status in long-living individuals

**DOI:** 10.1186/s12979-015-0054-8

**Published:** 2015-12-15

**Authors:** Francesco Villa, Alberto Malovini, Albino Carrizzo, Chiara C. Spinelli, Anna Ferrario, Anna Maciąg, Michele Madonna, Riccardo Bellazzi, Luciano Milanesi, Carmine Vecchione, Annibale A. Puca

**Affiliations:** Istituto di Tecnologie Biomediche, Consiglio Nazionale delle Ricerche, 20090 Segrate, MI Italy; Dipartimento di Ingegneria Industriale e Informatica, Università di Pavia, 27100 Pavia, Italy; IRCCS Neuromed - Parco Tecnologico, 86077 Pozzilli, IS Italy; IRCCS MultiMedica, 20138 Milan, Italy; Dipartimento di Medicina e Chirurgia, Università degli Studi di Salerno, Via Giovanni Paolo II, 132, 84084 Fisciano, SA Italy

**Keywords:** BPIFB4, Methylation, CD34, Vascular ageing

## Abstract

**Background:**

People that reach extreme ages (Long-Living Individuals, LLIs) are object of intense investigation for increase/decrease of genetic variant frequencies, genetic methylation levels, protein abundance in serum and tissues. The aim of these studies is the discovery of the mechanisms behind LLIs extreme longevity and the identification of markers of well-being. We have recently associated a BPIFB4 haplotype (LAV) with exceptional longevity under a homozygous genetic model, and identified that CD34^+^ of LLIs subjects express higher BPIFB4 transcript as compared to CD34^+^ of control population. It would be of interest to correlate serum BPIFB4 protein levels with exceptional longevity and health status of LLIs.

**Methods:**

Western blots on cellular medium to detect BPIFB4 secretion in transfected HEK293T cells with plasmid carrying BPIFB4 and ELISA on LLIs serum to detect BPIFB4 levels.

**Results:**

Here we show that BPIFB4 is a secreted protein and its levels are increased in serum of LLIs, and high BPIFB4 levels classify their health status.

**Conclusions:**

Serum BPIFB4 protein levels classify longevity and health status in LLIs. Further studies are required to evaluate the possible role of BPIFB4 in monitoring disease progression.

## Background

In the third millennium average life expectancy is 78 years for males and 83 years for females in industrialized countries, while at the beginning of the 20th century it was almost 20 years shorter [1]. Thus, the Long-Living Individuals (LLIs, >95 years of age) recruited in the 21st century reached around 30 years more than the average life expectancy of their cohorts. Among the LLIs some were able to escape illness (unaffected LLIs) while others survived to age-related diseases (cancer, diabetes, cardiovascular disease, or stroke) got at younger ages (affected LLIs) [2]. One of the principal factor in determining health during ageing is the state of the vascular system that is the responsible for provisioning all the body areas with nutrients and oxygen [3]. The aging process is associated with a reduction in NO availability in the endothelial district and this decrement triggers a progressive decline of the entire vascular system [4].

Our recent multi-step genetic analysis of Italian (the screening set),[5] and US and German LLIs (replication sets) and relative control populations ,[6–8] identified a variant in BPIFB4, which transcript showed to be down-regulated during aging and high in CD34^+^ of LLIs and the codified protein (LAV-BPIFB4) to be a powerful boost for endothelial vasorelaxation and revascularization, two functions lost during aging and cause of human frailty [9]. Furthermore, we have demonstrated that BPIFB4 is beneficial to cellular homeostasis by showing that: a) LAV-BPIFB4 potentiates eNOS activation; b) BPIFB4 isoforms cellular overexpression activates stress response (upregulation of heat shock proteins) and proteostasis (translation, ribosomal biogenesis and snoRNA/scaRNAs involved in genomic integrity), as also shown by reduction of eIF2alpha phosphorylation, thus activating protein synthesis.

Based on our previous results, we envisioned that serum levels of BPIFB4 could correlate with the ability to reach extreme ages and with LLIs health status.

## Results and discussion

**Fig. 1:**
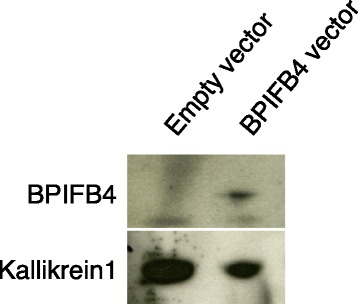
Analyses of the secretion of BPIFB4. The figure shows a Western blot analyses of the presence of BPIFB4 in medium of HEK293T cells transfected with Empty vector or BPIFB4-encoding plasmid. The evaluation of kallikrein 1 levels is used for normalization of protein content of the medium

To evaluate if BPIFB4 was secreted and detectable in serum, we first evaluated the medium of HEK293T cells transfected with BPIFB4-encoding plasmid (Fig. 1). Indeed, we found abundant BPIFB4 in the medium, confirming its extracellular secretion.

**Fig. 2:**
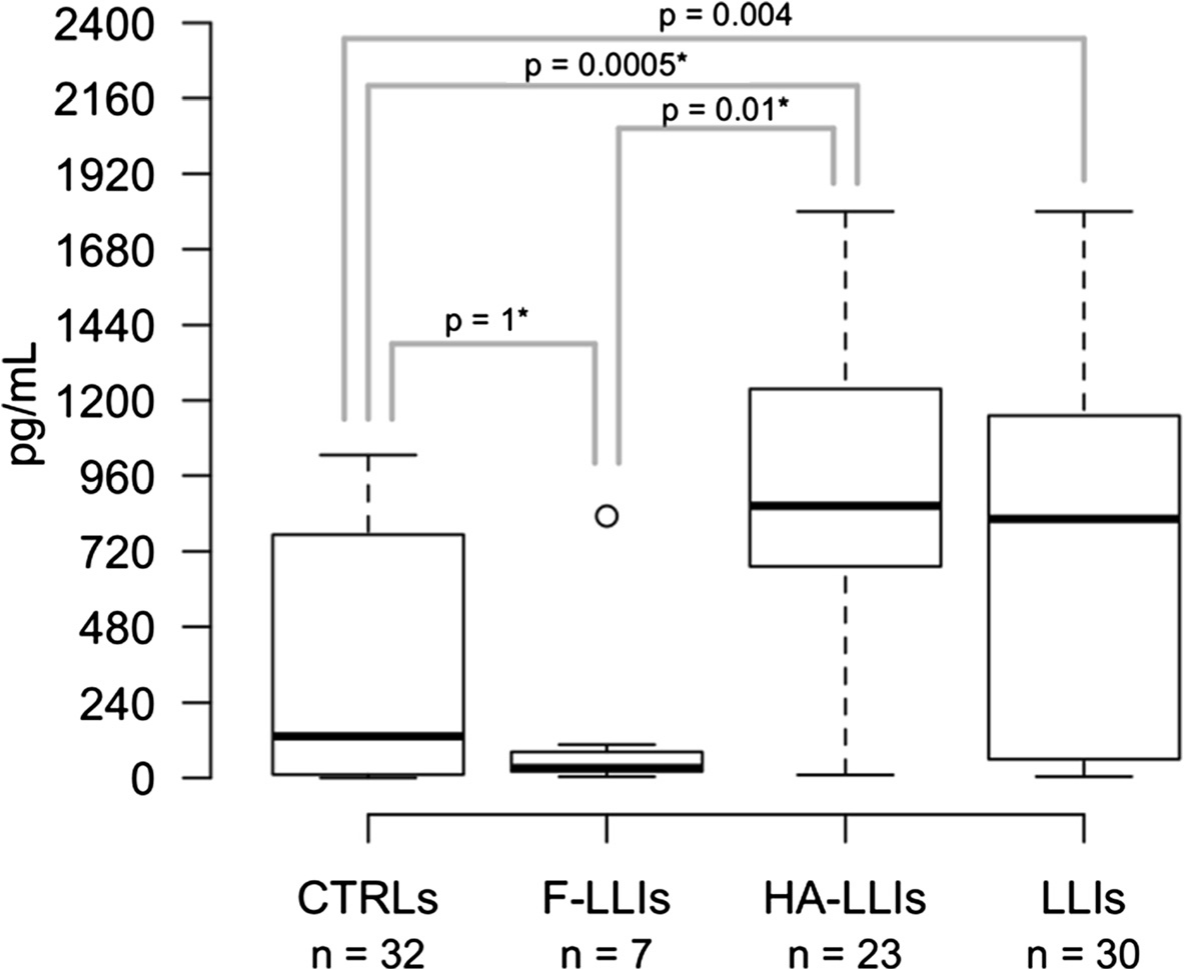
Boxplot describing the BPIFB4 level in human serum by sub-phenotypes. The boxplots show the concentration of BPIFB4 protein detected. Controls (CTRLs, *n* = 32); frail long-living individuals affected with cancer, diabetes, cardiovascular disease, or stroke (F-LLIs, *n* = 7); healthy-aged long-living individuals (HA-LLIs, *n* = 23); all long-living individuals (LLIs, *n* = 30). Each boxplot describes: i) the lower bound of the non – outliers range; ii) the 25th percentile; iii) the 50th percentile (median value); iv) the 75th percentile; v) the upper bound of the non – outliers range of the BPIFB4 distribution. Each dot represents an outlier value with respect to the corresponding distribution. *P-*values were estimated by the non-parametric Wilcoxon Rank – Sum test. Bonferroni correction (*) was applied when testing for differences in terms of protein concentrations between F-LLIS, HA-LLIs and CTRLs

We then used ELISA to evaluate the amount of the protein in sera from middle-aged individuals (age range: 45–59 years, *n* = 32) and from LLIs (>94 years, *n* = 30) (see Table 1 for details). We found the serum BPIFB4 level to be significantly higher in LLIs compared to controls (median ELISA quantifications in LLIs = 823.76, IQR = 69.91–1146.35 *vs.* median ELISA quantifications in CTRLs = 132.74, IQR = 11.25–751.32, p - value = 0.004, Fig. 2), pointing to BPIFB4 as a protein associated with longevity.

**Table 1:** LLIs groups description

Group	n	Median age (IQR)	Age range	Females (%)	Familiarity for longevity (%)
F-LLIs	7	97 (96–99)	94–100	71.43	14.29
HA-LLIs	23	96 (95–97)	94–103	69.57	13.04
LLIs	30	96 (95–98)	94–103	70.00	13.33

*F-LLIs* frail long-living individuals affected with cancer, *diabetes* cardiovascular disease, or stroke; *HA-LLIs* healthy-aged long-living individuals, *LL*Is all long-living individuals

Then we looked into the LLIs population to test whether BPIFB4 serum levels could discriminate between their healthy statuses. We analyzed different variables, which included age, familiarity for longevity trait, affection status (defined as presence or absence of medical history for cancer, diabetes, cardiovascular disease, or stroke) and gender. Of the analyzed variables, ELISA quantifications were significantly lower in affected LLIs (or frail LLIs, F-LLIs, *n* = 7) with respect to the rest of the LLIs (or healthy aged individuals, HA-LLIs, *n* = 23) (median ELISA quantifications in F-LLIs = 31.21, IQR = 20.56–82.78 *vs.* median ELISA quantifications in HA-LLIs = 865.22, IQR = 672.30–1236.43, *p* = 0.004, Bonferroni adjusted *p* = 0.01, Fig. 2). No statistically significant difference in terms of age, familiarity for longevity trait or gender was observed between F-LLIs and HA-LLIs (*p* > 0.05). We further observed that the protein concentration was significantly higher in HA-LLIs compared to CTRLs (median ELISA quantifications in HA-LLIs = 865.22, IQR = 672.30–1236.43 *vs.* median ELISA quantifications in CTRLs = 132.74, IQR = 11.25–751.32, *p* = 0.0002, Bonferroni adjusted *p* = 0.0005). No statistically significant difference was observed in terms of protein concentration between F-LLIs and CTRLs (median ELISA quantifications in F-LLIs = 31.21, IQR = 20.56–82.78vs. median ELISA quantifications in CTRLs = 132.74, IQR = 11.25–751.32, *p* = 0.570, Bonferroni adjusted *p* = 1).

**Fig. 3:**
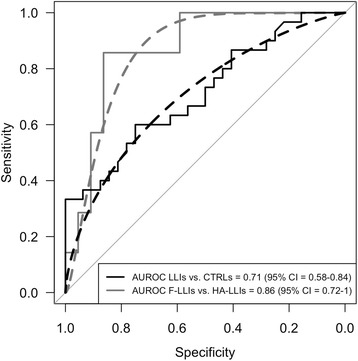
ROC Curves. Smoothed (dashed lines) and not smoothed (continuous lines) Receiver Operating Characteristic (ROC) curves and Area Under the Receiver Operating Characteristic (AUROC) corresponding to the ELISA quantifications in discriminating LLIs from CTRLS and F-LLIs from HA-LLIs respectively. 95 % CI = 95 % Confidence Interval. AUROC estimates and 95 % CI refer to the two not smoothed curves

Figure 3 shows the ROC curve and the AUROC corresponding to the ELISA quantification in discriminating LLIs from CTRLs (AUROC = 0.71, 95 % CI = 0.58–0.84) and F-LLIs from HA-LLIs (AUROC = 0.86, 95 % CI = 0.72–1).

**Fig. 4:**
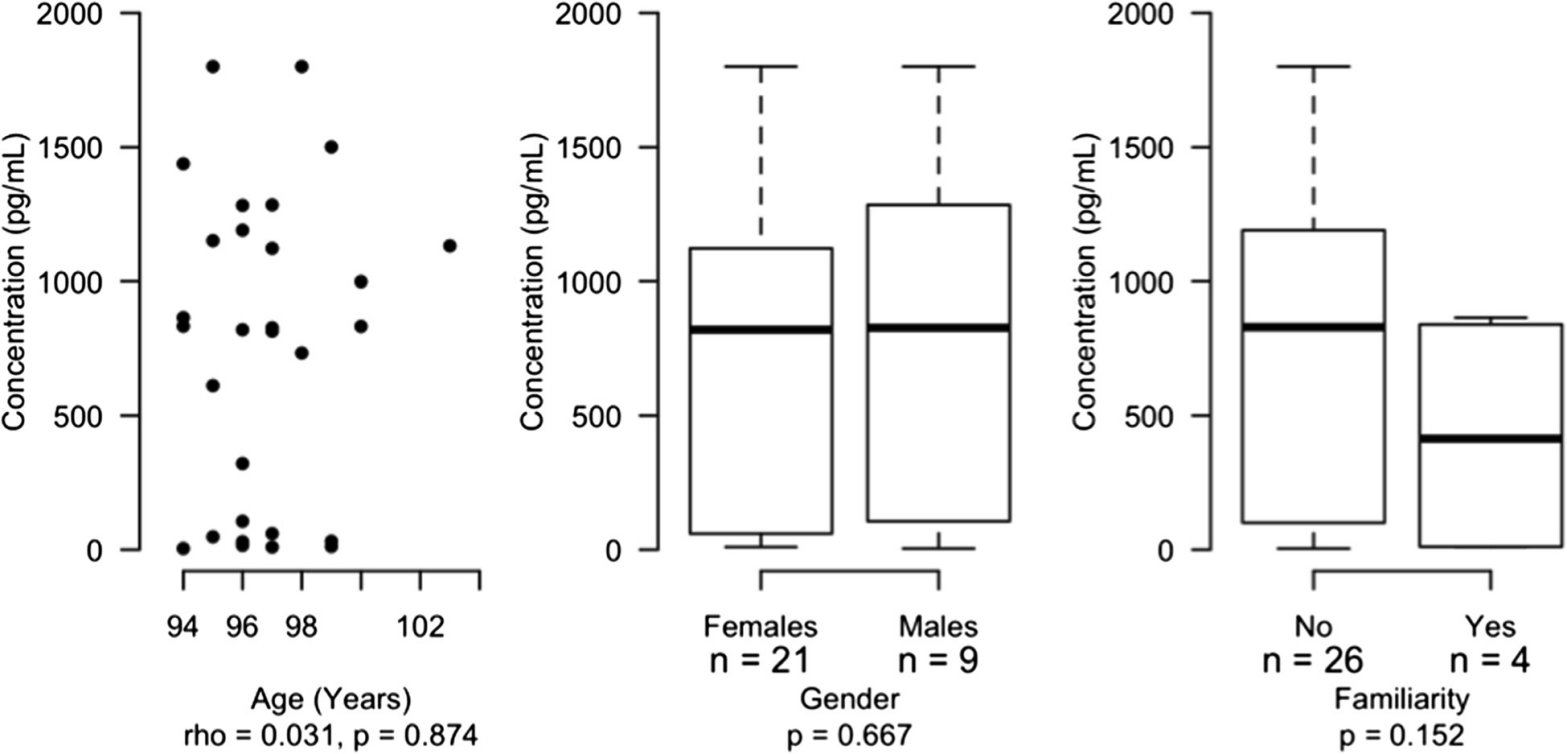
Plots Scatterplot describing the correlation between age and ELISA quantifications and boxplots reporting the ELISA quantifications distribution by gender and familiarity. Rho = Spearman correlation coefficient

No other variable was associated to statistically significant variations in terms of ELISA quantifications (*p* > 0.05 Fig. 4).

The power of the ELISA quantifications and of the combination of ELISA quantifications plus other clinical variables (age, gender and familiarity for longevity trait) in discriminating F-LLIs from HA-LLIS were assessed by stepwise logistic regression coupled with a Leave One Out (LOO) cross validation schema [10] on a subset of 29 LLIs showing complete data for all covariates (n F-LLIS = 7, n H-LLIS = 22). According to this resampling strategy, three models (*clinical covariates based model* (age + gender + familiarity); *clinical covariates and ELISA quantifications based model* (ELISA quantifications + age + gender + familiarity) and *ELISA quantifications based model* (ELISA quantifications)) were learned on the each training set using the LLIs disease status as outcome (F-LLIs were coded *1*, HA-LLIs were coded *0*) and the generalization capability of the set of features selected by the stepwise regression tested on the external test set. Results are reported in Table 2 and they confirm that ELISA quantification was the strongest parameter to correctly discriminate F-LLIs from HA-LLIs, since no other combination of clinical covariates or clinical covariates plus ELISA quantifications was able to outperform its classification capability. However, these results should be cautiously interpreted due to the small sample size of our analysis and due to the lack of an independent cohort of individuals to be used to confirm the generalization capability of the BPIFB4 serum levels as marker of healthy aging.

**Table 2:** Discriminative performances obtained by different combinations of covariates

Coefficient	Clinical Covariates	ELISA Quantification	ELISA Quantification + Clinical Covariates
AUROC	0	0.74	0.68
MCC	NaN	0.67	0.25
Sensitivity	0	0.86	0.43
Specificity	1	0.86	0.82
PPV	NaN	0.67	0.43
NPV	0.76	0.95	0.82
F-Measure	NaN	0.75	0.43

*AUROC* Area Under the ROC Curve, *MCC* Mattew’s Correlation Coefficient, *PPV* Positive Predictive Value, *NPV* Negative Predictive Value

We have recently found a potential link between BPIFB4 and exceptional longevity by analyzing more than 3000 LLIs individuals and 2000 controls of different recruitment efforts (Italian, US and German). We have identified a homozygous enrichment of the longevity associated variant haplotype (LAV) in LLIs indicating a possible protective role of this genotype [9, 11]. Indeed, further studies have shown that the BPIFB4 transcript was high in CD34^+^ cells of LLIs, corroborating a possible protective role of the protein. Such a role was further investigated in vitro by overexpressing BPIFB4 variants and in vivo by injecting mice and rats with AAV carrying different isoforms of the BPIFB4 gene. In vitro studies pointed to a role of the protein in survival processes, such as stress response, proteostasis, genomic integrity, and in vivo experiments of role of LAV in potentiating eNOS activation, endothelial function, revascularization after induced ischemia. All these aspects are lost during aging and we hypothesize that can be kept active in LLIs by higher expression of the BPIFB4 protein. Indeed, here we describe that BPIFB4 is secreted and ELISA detected higher BPIFB4 levels in serum of LLIs as compared to young controls, that BPIFB4 serum levels can be used to classify the LLIs health status (affected versus unaffected) and that classification was not improved by including in the analysis familiarity for longevity and age. Recently, Heyn et al. [12] generated methylation profiles of 485,577 highly informative CpG sites (Illumina Infinium Human Methylation 450 BeadChip) and showed a list of CpG hypomethylated (*N* = 1920), which included cg04087207 in the first exon of BPIFB4, indicating its possible overexpression. Thus, the analysis, which was performed in CD4^+^ cells of 19 LLIs in comparison to 19 newborns (NBs), would support our observation that BPIFB4 is more abundant in LLIs as compared to young controls.

## Conclusions

Our results, which describe the detection of high levels of BPIFB4 protein in LLIs serum, further support the hypothesis of an important role of the protein in exceptional longevity. BPIFB4 levels could be adopted as biomarkers of health status in LLIs.

Further studies will evaluate the potential use of BPIFB4 levels for following health of individuals with pathologies.

## Methods

### BPIFB4 overexpression and western blot analyses

HEK293T cells were grown in culture medium (DMEM, 10 % fetal bovine serum, 2 mM L-glutamine, 100 U penicillin/0.1 mg/ml streptomycin) and transfected with pRK5 vector encoding BPIFB4, or with an empty plasmid, using Lipofectamine 2000 (Life Technologies) according to the manufacturer’s protocol, in triplicates. 24 h after transfection, the medium is been changed and finally collect 48 h after the change. Protein content of medium is been concentrated using Amicon Ultra columns (Merck Millipore) and analyzed by Western blot. Protein were separated on 10 % SDS-PAGE at 100 V for 1 h or on 4–12 % SDS-PAGE at 100 V for 2 h and then transferred to a nitrocellulose or PVDF membrane. The membranes were incubated overnight with primary antibodies of anti-BPIFB4 (Abcam, rabbit pAb, 1:200) and anti-kallikrein 1 (Abcam, mouse pAb, 1:1000). After a triple wash, membranes were incubated for 1 or 2 h with the secondary antibody (Amersham Life Science, horseradish peroxidase-linked anti-rabbit IgG or anti-mouse IgG, 1:3000). The membranes were then washed four times and specific protein bands were detected with ECL Prime chemiluminescent agents (Amersham Life Science).

### Samples recruitment and groups identification

All the samples were recruited in the same period and the sera collection were performed by centrifugation of serum separator tubes (SST) and immediately stored at −80 °C. The anamnestic information were collected at the same time and the frail LLIs and the healthy aged LLIs groups were discriminated by the presence of important systemic diseases.

All subjects donated blood samples for DNA study and gave written informed consent to the study, which was approved by Ethical Committee. The study was conducted in accordance with the ethical principles that have their origins in the Declaration of Helsinki.

### Measurement of BPIFB4 in human serum

Quantitative determination of BPIFB4 in the sera of 32 control subjects and 30 LLIs was performed in triplicate with commercial ELISA (code: CSB-EL003694HU, CUSABIO, P.R. China) following the manufacturer’s protocol. The absorbance of the assay was detected using a Microplate Reader Synergy 2, BioTek.

### Statistical analyses

Since ELISA quantifications deviated significantly from the normal distribution (Shapiro-Wilk Test *p-value* < 0.01), non-parametric univariate tests were applied. The correlation between age and ELISA quantifications was tested by the Spearman Correlation Test, while differences in terms of ELISA quantifications between binary variables (i.e., disease condition, gender and familiarity) were tested by the Wilcoxon Rank Sum Test. Stepwise logistic regression coupled with Leave One Out (LOO) cross validation strategy was applied to identify variables modulating the probability of disease in LLIs (F-LLIs were coded *1*, HA-LLIs were coded *0*) [10]. According to the LOO strategy, stepwise logistic regression models were learned on each training set independently, starting from the following 3 sets of covariates:

- *clinical covariates based model: age + gender + familiarity*
- *clinical covariates and ELISA quantifications based model: ELISA quantifications + age + gender + familiarity*
- *ELISA quantifications based model: ELISA quantifications*

The models learned on each training set were then used to predict the probability of being F-LLI of the individual not included in the learning phase. Probabilities of being F-LLIs were discretized using a cut-off of 0.5 (probability ≥0.5 = F-LLI condition, probability <0.5 = HA-LLI).

By defining a F-LLI correctly classified as a true positive (TP), a HA-LLI correctly classified as a true negative (TN), a HA-LLI record erroneously classified as a false positive (FP) and a F-LLI erroneously classified as a false negative (FN), the discriminative performances of each model were expressed in terms of sensitivity (sensitivity = TP / (TP + FN)), specificity (specificity = TN / (TN + FP)), positive predictive value (PPV = TP / (TP + FP)), negative predictive value (NPV = TN / (TN + FN)), Matthews correlation coefficient (MCC = (TP * TN - FP * FN) / sqrt((TP + FP) * (TP + FN) * (TN + FP) * (TN + FN))) and F-Measure (2 * ((PPV * sensitivity) / (PPV + sensitivity))).

Quantitative distributions are described by median (25–75th percentiles), p-values are two-sided. All statistical analyses were performed by the R statistical software (www.r-project.org) [13].

## Abbreviations

F-LLIs: frail LLIs
HA-LLIs: healthy aged LLIs
IQR: interquartile range
LAV-BPIFB4: longevity associated variant –BPIFB4
LLIs: long-living individuals
NO: nitric oxide

## References

[1] Oeppen J, Vaupel JW (2002). Demography. Broken limits to life expectancy. Science.

[2] Evert J, Lawler E, Bogan H, Perls T (2003). Morbidity profiles of centenarians: survivors, delayers, and escapers. J Gerontol A Biol Sci Med Sci.

[3] Puca AA, Carrizzo A, Ferrario A, Villa F, Vecchione C (2012). Endothelial nitric oxide synthase, vascular integrity and human exceptional longevity. Immun Ageing.

[4] Puca AA, Carrizzo A, Villa F, Ferrario A, Casaburo M, Maciag A (2013). Vascular ageing: the role of oxidative stress. Int J Biochem Cell Biol.

[5] Malovini A, Illario M, Iaccarino G, Villa F, Ferrario A, Roncarati R (2011). Association study on long-living individuals from Southern Italy identifies rs10491334 in the CAMKIV gene that regulates survival proteins. Rejuvenation Res.

[6] Geesaman BJ, Benson E, Brewster SJ, Kunkel LM, Blanche H, Thomas G (2003). Haplotype-based identification of a microsomal transfer protein marker associated with the human lifespan. Proc Natl Acad Sci U S A.

[7] Nebel A, Kleindorp R, Caliebe A, Nothnagel M, Blanche H, Junge O (2011). A genome-wide association study confirms APOE as the major gene influencing survival in long-lived individuals. Mech Ageing Dev.

[8] Novelli V, Viviani Anselmi C, Roncarati R, Guffanti G, Malovini A, Piluso G (2008). Lack of replication of genetic associations with human longevity. Biogerontology.

[9] Villa F, Carrizzo A, Spinelli CC, Ferrario A, Malovini A, Maciag A et al. Genetic Analysis Reveals a Longevity-Associated Protein Modulating Endothelial Function and Angiogenesis. Circ Res. 2015;117(4):333-45. doi:CIRCRESAHA.117.305875 [pii] 10.1161/CIRCRESAHA.117.305875. 10.1161/CIRCRESAHA.117.305875 PMC5496930 26034043

[10] Hastie T, Tibshirani R, Friedman J. The Elements of Statistical Learning: Data Mining, Inference, and Prediction. Second Edition ed. Springer; USA, 2009.

[11] Kraehling JR, Sessa WC (2015). Enhanced eNOS Activation as the Fountain of Youth for Vascular Disease: Is BPIFB4 What Ponce de Leon Was Looking For?. Circ Res.

[12] Heyn H, Li N, Ferreira HJ, Moran S, Pisano DG, Gomez A (2012). Distinct DNA methylomes of newborns and centenarians. Proc Natl Acad Sci U S A.

[13] R Development Core Team (2009). R: A language and environment for statistical computing.

